# Cardiotoxicity during long-term trastuzumab use in patients with HER2-positive metastatic breast cancer: who needs cardiac monitoring?

**DOI:** 10.1007/s10549-020-06039-w

**Published:** 2021-01-04

**Authors:** N. I. Bouwer, T. G. Steenbruggen, J. van Rosmalen, H. N. Rier, J. J. E. M. Kitzen, M. L. van Bekkum, A. J. Ten Tije, P. C. de Jong, J. C. Drooger, C. Holterhues, C. H. Smorenburg, M. J. M. Kofflard, E. Boersma, G. S. Sonke, M.-D. Levin, A. Jager

**Affiliations:** 1 Department of Internal Medicine, Albert Schweitzer Hospital, 3300 AK Dordrecht, South-Holland The Netherlands; 2 Department of Cardiology, Albert Schweitzer Hospital, Dordrecht, The Netherlands; 3 Department of Medical Oncology, The Netherlands Cancer Institute, Amsterdam, The Netherlands; 4 Department of Biostatistics, Erasmus MC, University Medical Centre Rotterdam, Rotterdam, The Netherlands; 5 Department of Medical Oncology, Erasmus MC, Cancer Institute, Rotterdam, The Netherlands; 6 Department of Medical Oncology, Reinier de Graaf Hospital, Delft, The Netherlands; 7 Department of Medical Oncology, Amphia Hospital, Breda, The Netherlands; 8 Department of Medical Oncology, Sint Antonius Hospital, Utrecht, The Netherlands; 9 Department of Medical Oncology, Ikazia Hospital, Rotterdam, The Netherlands; 10 Department of Medical Oncology, Haga Hospital, The Hague, The Netherlands; 11 Department of Cardiology, Erasmus MC, University Medical Centre Rotterdam, Rotterdam, The Netherlands

**Keywords:** HER2-positive metastatic breast cancer, Trastuzumab treatment, Cardiotoxicity, LVEF monitoring, Screening for cardiotoxicity

## Abstract

**Purpose:**

Patients with HER2-positive metastatic breast cancer (MBC) usually receive many years of trastuzumab treatment. It is unknown whether these patients require continuous left ventricular ejection fraction (LVEF) monitoring. We studied a real-world cohort to identify risk factors for cardiotoxicity to select patients in whom LVEF monitoring could be omitted.

**Methods:**

We included patients with HER2-positive MBC who received > 1 cycle of trastuzumab-based therapy in eight Dutch hospitals between 2000 and 2014. Cardiotoxicity was defined as LVEF < 50% that declined > 10%-points and was categorized into non-severe cardiotoxicity (LVEF 40–50%) and severe cardiotoxicity (LVEF < 40%). Multivariable Cox and mixed model analyses were performed to identify risk factors associated with cardiotoxicity. Additionally, we explored the reversibility of cardiotoxicity in patients who continued trastuzumab.

**Results:**

In total, 429 patients were included. Median follow-up for cardiotoxicity was 15 months (interquartile range 8–31 months). The yearly incidence of non-severe + severe cardiotoxicity in the first and second year was 11.7% and 9.1%, respectively, which decreased thereafter. The yearly incidence of severe cardiotoxicity was low (2.8%) and stable over time. In non-smoking patients with baseline LVEF > 60% and no cardiotoxicity during prior neoadjuvant/adjuvant treatment, the cumulative incidence of severe cardiotoxicity was 3.1% after 4 years of trastuzumab. Despite continuing trastuzumab, LVEF decline was reversible in 56% of patients with non-severe cardiotoxicity and in 33% with severe cardiotoxicity.

**Conclusions:**

Serial cardiac monitoring can be safely omitted in non-smoking patients with baseline LVEF > 60% and without cardiotoxicity during prior neoadjuvant/adjuvant treatment.

**Supplementary Information:**

The online version of this article (10.1007/s10549-020-06039-w) contains supplementary material, which is available to authorized users.

## Introduction

Trastuzumab is a monoclonal antibody targeting the human epidermal growth factor receptor 2 (HER2) that has greatly improved the outcome of patients with HER2-positive breast cancer in both the primary and metastatic setting [1–3]. Trastuzumab toxicity is generally mild, although left ventricle ejection fraction (LVEF) decline (cardiotoxicity) is a well-known side effect that is mostly seen in combination with concurrent or sequential anthracycline treatment [3]. Regular LVEF monitoring at a 3-monthly interval is therefore recommended during 1 year of neoadjuvant and/or adjuvant trastuzumab treatment; however, during metastatic treatment no specific time interval of LVEF monitoring is recommended [4]. Since the median overall survival of patients with HER2-positive metastatic breast cancer (MBC) is well over 4 years with continuous use of trastuzumab, the cumulative burden of LVEF monitoring can be high [5, 6]. The incidence of cardiotoxicity during long-term treatment for HER2-positive MBC, however, is not well-known and neither are risk factors for trastuzumab-associated cardiotoxicity in this setting.

Two studies investigated cardiotoxicity over time during trastuzumab treatment in patients with MBC [7, 8]. The first study found a cumulative incidence of cardiotoxicity of 12.7% and 28.5% after 1 and 3 years of trastuzumab use, respectively [7]. They defined cardiotoxicity as  LVEF decline > 20%-points from baseline or LVEF < 50% or symptoms of congestive heart failure. The LVEF recovered in a vast majority of the patients (84%) after discontinuation of trastuzumab with or without cardio-protective treatment. However, reversibility of cardiotoxicity after continuation of trastuzumab has not been described yet. A second study observed a cumulative incidence of cardiotoxicity of 5.3% after 3 years of trastuzumab use. However, they used a composite endpoint that included myocardial ischaemia, heart failure, rhythm disorder, and other cardiac diseases [8]. Data on long-term sequelae were not available. Lastly, risk factors for developing cardiotoxicity during long-term trastuzumab treatment could be similar to those causing cardiotoxicity during 1 year of trastuzumab treatment [9–11]; however, this has not been investigated yet.

Therefore, we studied cardiotoxicity during long-term trastuzumab treatment in patients with HER2-positive MBC in an observational historic multicentre cohort study and risk factors associated with cardiotoxicity in this setting to select patients in whom LVEF monitoring could be omitted. Additionally, we evaluated the reversibility of cardiotoxicity in patients who continued trastuzumab.

## Methods

### Patients and data collection

We included patients with HER2-positive MBC who received > 1 cycle of trastuzumab-based treatment in one of eight participating Dutch hospitals between January 2000 and December 2014, as described before [12]. Patients were identified using the Netherlands Cancer Registry. Patients were excluded in case no baseline LVEF measurement was available within 30 days before the first trastuzumab administration for MBC, baseline LVEF < 50%, no follow-up LVEF measurements were available during trastuzumab use, and in case of incomplete clinical data in the medical records.

Trained investigators systematically retrieved data on patient and tumour characteristics, treatment, and LVEF measurements from medical records. Medical Ethics Commission of all participating hospitals approved this comprehensive data collection.

### Endpoints

We defined non-severe + severe cardiotoxicity and severe cardiotoxicity based on guidelines of the European Society of Cardiology (ESC) [13] and the European Society of Medical Oncology (ESMO) [4], respectively. First, *non-severe* + *severe cardiotoxicity* is defined as (1) LVEF decline > 10%-points from baseline and LVEF < 50% measured with multigated acquisition (MUGA) scan or (2) decline from good/normal cardiac function to at least mild cardiac dysfunction and at least mild cardiac dysfunction measured with echocardiography if MUGA was not available. After a decline in LVEF found with MUGA scan, in some cases this was followed by an echocardiography to exclude false negative low LVEF measurements. In case both investigations were performed, echocardiography was used to define cardiotoxicity. Second, *severe cardiotoxicity* is defined as (1) LVEF < 40% measured with MUGA scan or (2) moderate or severe cardiac dysfunction measured with echocardiography if MUGA was not available [13]. Since patients with LVEF < 50% at baseline were excluded, patients with LVEF < 40% had by definition a LVEF decline of > 10%-points compared to baseline. Lastly, *non-severe cardiotoxicity* was defined as (1) LVEF < 50% but > 40% or mild cardiac dysfunction measured with echocardiography. The time to non-severe + severe cardiotoxicity, non-severe cardiotoxicity or severe cardiotoxicity was calculated from start of trastuzumab treatment for MBC to the first occurrence of non-severe + severe cardiotoxicity.

For the analyses of reversibility, non-severe + severe cardiotoxicity was categorized into non-severe cardiotoxicity and severe cardiotoxicity. Reversibility of cardiotoxicity was defined as any LVEF increase to a value < 5% below baseline, partially reversibility as any absolute LVEF increase ≥ 10% from nadir and to a value > 5% below baseline, and irreversibility as any absolute LVEF increase < 10% from nadir and to a value > 5% below baseline [14]. The frequency of LVEF measurements was determined by the treating physician.

### Statistical analyses

Continuous variables are presented as medians with interquartile range (IQR) for non-normal distribution, and as means with standard deviations for normal distribution. Categorical variables are presented as percentages.

Median follow-up for cardiotoxicity was calculated from start of trastuzumab for MBC until 6 months after last trastuzumab dose or until last LVEF measurement, whichever came first. Discontinuation of trastuzumab treatment was defined as any stop or interruption of trastuzumab treatment. The yearly incidence of cardiotoxicity was investigated. Patients were at risk for cardiotoxicity when receiving LVEF measurements during trastuzumab treatment. After the first development of non-severe cardiotoxicity, patients were no longer at risk for non-severe cardiotoxicity. However, these patients were still at risk for severe cardiotoxicity. After developing severe cardiotoxicity, patients were no longer at risk for any type of cardiotoxicity.

We used univariable and multivariable Cox proportional hazards (PH) analyses to determine which baseline variables were associated with non-severe + severe cardiotoxicity and severe cardiotoxicity, and to find a group of patients at low risk of cardiotoxicity. All variables were determined at start of trastuzumab treatment for MBC. Independent variables statistically significant at 0.10 level in univariable analysis or known risk factors for cardiotoxicity from literature were included in the multivariable analysis. The Cox PH assumption was verified using the Schoenfeld residuals test and was not violated. Multivariable cause-specific Cox PH models were built with similar variables to investigate potential competing risk from death with non-severe + severe cardiotoxicity or severe cardiotoxicity. In this analysis, patients are censored in case of death due to any cause.

The following variables were included in the analyses: age, BMI, hypertension, diabetes mellitus, smoking, history of cardiac disease, baseline LVEF < 60%, cardiotoxicity during prior neoadjuvant/adjuvant treatment with trastuzumab and/or anthracycline, prior cumulative anthracycline exposure, radiation exposure to the breast. BMI was categorized in BMI < 25 kg/m^2^, 25–30 kg/m^2^, and > 30 kg/m^2^. Hypertension was defined as a history of systolic blood pressure > 130 mmHg or diastolic blood pressure > 80 mmHg or the use of antihypertensive medication [15]. History of cardiac disease was defined as the history of either arrhythmia, cardiac valve deficiency, cardiomyopathy or coronary artery disease. Cardiotoxicity during prior treatment was defined LVEF decline > 10%-points to a LVEF < 50% during neoadjuvant/adjuvant treatment with trastuzumab and/or anthracycline. Prior cumulative anthracycline exposure was defined as the number of courses anthracycline before trastuzumab in palliative setting. Radiation exposure was categorized in left-sided, right-sided or unknown side. Last, de novo metastatic breast cancer was defined as metastatic disease at time of diagnosis or development of metastases within 3 months of diagnosis.

For the multivariable Cox PH analyses, missing information on diabetes mellitus, hypertension, smoking, history of cardiac disease, radiotherapy side and cardiotoxicity during prior neoadjuvant/adjuvant treatment with trastuzumab and/or anthracycline was imputed using fully conditional specification with 100 imputations. Estimates were pooled over imputed data sets using Rubin’s rules. A sensitivity analysis with a complete case analysis was conducted to investigate robustness of the imputation procedure. In the cause-specific Cox PH analyses, missing values were imputed using substantive model compatible fully conditional specification with 100 imputations. Additionally, sensitivity analyses were performed by investigating the number of LVEF measurements as a risk factor for cardiotoxicity in a Cox PH analysis to investigate detection bias. LVEF measurements up until the development of cardiotoxicity were taken into account in these analyses.

To study the relation between the independent variables and continuous LVEF measurements during total follow-up, linear mixed effects model (LMM) analysis was conducted. Risk factors that are related to a LVEF decline, but not with the definition of cardiotoxicity, were investigated in this analysis. In addition to the variables used in the Cox PH analysis, this model allows to investigate time varying variables, namely the cumulative trastuzumab exposure at each LVEF measurement calculated from start trastuzumab to each LVEF measurement (in months). The fit of the LMM was adjusted by the non-linear curve observed from the predicted values plot (data not shown) [16]. The effect of time since start trastuzumab was modelled using restricted cubic splines, with the number of knots (4) chosen using information criteria. Random intercept and random slope of time since start of trastuzumab were included to account for within-patient correlations between repeated measurements.

Inverse Kaplan–Meier curves stratified for significant risk factors were used to investigate the cumulative incidence of non-severe + severe cardiotoxicity and severe cardiotoxicity for the number of significant risk factors from the Cox PH analysis.

Data analyses were performed using SPSS (version 24.0) and R (version 3.4.3), in particular the packages “lme”, “splines”, “JointAI”, “smcfcs” and “mice”.

## Results

### Patient characteristics

Between January 2000 and December 2014, 745 patients with HER2-positive MBC were identified in the eight participating Dutch hospitals. After excluding patients who had no baseline LVEF measurement (*n* = 193), a baseline LVEF measurement < 50% (*n* = 41), no LVEF measurement during trastuzumab treatment (*n* = 55), or in whom no additional data collection was possible (*n* = 18), 429 patients were eligible for current analyses (Supplementary Figure S1). In general, no difference was observed between included and excluded patients, except for a lower percentage of patients receiving prior neoadjuvant/adjuvant trastuzumab in the latter (Supplementary Table S1). Patient and treatment characteristics are shown in Table 1. Of all patients, 311 (72%) had metachronous distant metastases and 118 (28%) had synchronous distant metastases at disease presentation.

**Table 1:** Baseline characteristics of all included patients (*n* = 429)

Clinical and treatment characteristics	No. (%), median [IQR]
Age (years)^a^	54 [46–61]
Hormonal receptor status^b^	
Positive	235 (55)
Negative	172 (40)
Unknown	22 (5)
Neoadjuvant/adjuvant chemotherapy	
No	213 (50)
Anthracyclines + trastuzumab	65 (15)
Anthracyclines without trastuzumab	120 (28)
Trastuzumab without anthracyclines	19 (4)
Other	12 (3)
Duration of neoadjuvant/adjuvant trastuzumab (months)	12 [12–12]
Prior cumulative anthracycline exposure (courses)^a,c,d^	0 [0–4]
Adjuvant radiotherapy	
No	171 (40)
Left side	126 (29)
Right side	100 (23)
Side unknown	32 (8)
Cardiotoxicity during prior neoadjuvant/adjuvant treatment with trastuzumab and/or anthracyclines	
No	329 (77)
Yes	15 (4)
Unknown	85 (20)
Baseline LVEF (%)^a^	
≥ 60	198 (46)
< 60	231 (54)
BMI (kg/m^2^)^a^	
< 25	164 (49)
25–30	130 (30)
> 30	44 (10)
Unknown	91 (21)
History of cardiac disease	
No	379 (88)
Yes	35 (8)
Unknown	15 (4)
Diabetes mellitus^a^	
No	387 (90)
Yes	28 (7)
Unknown	14 (3)
Hypertension^a^	
No	314 (73)
Yes	98 (23)
Unknown	17% (4)
Hypercholesterolemia^a^	
No	210 (49)
Yes	37 (9)
Unknown	182 (42)
Smoking^a^	
No	203 (47)
Yes	90 (21)
Unknown	136 (32)

IQR interquartile range, *LVEF* left ventricle ejection fraction, *BMI* body mass index, *MUGA* scan, multigated acquisition scan, *MRI* magnetic resonance imaging, *M*BC metastatic breast cancer

^a^At start of palliative trastuzumab treatment; for definition see Methods section

^b^Estrogen and progesterone receptor positivity was defined as ≥ 10% positive nuclear staining [17]

^c^Number of courses before palliative trastuzumab treatment

^d^Could consist of doxorubicin or epirubicin courses

Patients were followed for cardiotoxicity with a median of 15 months (IQR 8–31). The median overall survival for all patients was 42 months (IQR 25–71). Median frequency of LVEF monitoring was 4 times annually (IQR 3–5) with a median total number of LVEF measurements of 4 during follow-up (IQR 2–7). Most commonly used cardiac imaging modality for LVEF assessment was the MUGA scan in 358 patients (83%). Echocardiography (4%) or CMR (0.2%) or a combination of both (13%) was performed less often.

### Incidence of non-severe + severe and severe cardiotoxicity over time

**Fig. 1:**
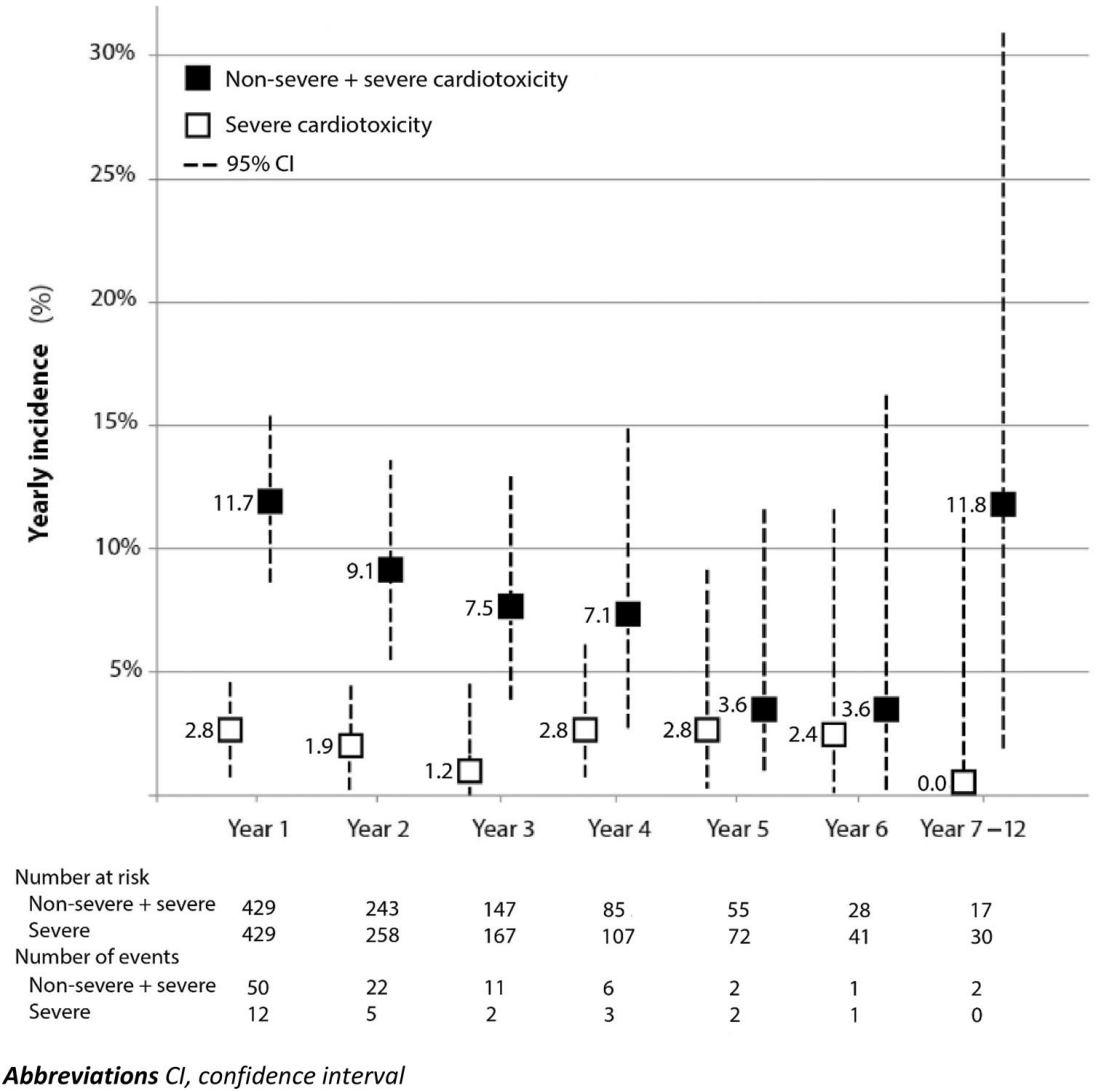
Yearly incidence of non-severe + severe cardiotoxicity and severe cardiotoxicity during long-term trastuzumab treatment

During total follow-up, 94 patients (22%) developed non-severe + severe cardiotoxicity. In the first year of trastuzumab treatment, the incidence of non-severe + severe cardiotoxicity was 11.7% (Fig. 1). The yearly incidence gradually decreased over the following years (i.e. 9.1% in year 2 to 3.6% in year 6). The median time to develop non-severe + severe cardiotoxicity from start of trastuzumab for MBC was 11 months (IQR 5–23).

In total, 25 patients (6%) developed severe cardiotoxicity. In the first year of trastuzumab treatment, the incidence of severe cardiotoxicity was 2.8% (Fig. 1). The yearly incidence of severe cardiotoxicity the next years remained stable (i.e. 1.9% in year 2 to 2.4% in year 6). The median time to severe cardiotoxicity was 10 months (IQR 6–25).

### Risk factors associated with non-severe + severe cardiotoxicity and severe cardiotoxicity

Risk factors independently associated with non-severe + severe cardiotoxicity were BMI > 30 kg/m^2^ (adjusted [a]HR 2.16, 95% CI 1.15–4.06), smoking (aHR 1.73, 95% CI 1.05–2.85), cardiotoxicity during prior neoadjuvant/adjuvant treatment with trastuzumab and/or anthracyclines (aHR 4.48, 95% CI 1.56–12.87). Prior neoadjuvant/adjuvant trastuzumab (aHR 0.38, 95% CI 0.18–0.82) was associated with less non-severe + severe cardiotoxicity (Table 2).

**Table 2:** Risk factors present at start of trastuzumab treatment for MBC associated with developing non-severe + severe cardiotoxicity

	Univariable Cox PH^a^			Multivariable Cox PH^a^		
	HR	95% CI	*p*-value	Adjusted HR	95% CI	*p*-value
Age (years)	1.01	0.99–1.02	0.574	1.01	0.99–1.03	0.254
BMI (kg/m^2^)						
< 25	Ref					
25–30	1.29	0.76–2.17	0.340	1.24	0.73–2.12	0.423
> 30	2.05	1.13–3.73	0.018	2.16	1.15–4.06	0.017
Hypertension	1.23	0.77–1.95	0.386			
Diabetes mellitus	1.20	0.55–2.64	0.651			
Smoking	1.89	1.13–3.16	0.016	1.73	1.05–2.85	0.031
History of cardiac disease	1.36	0.71–2.60	0.352			
Baseline LVEF (%)						
≥ 60	Ref					
< 60	1.60	1.05–2.44	0.030	1.52	0.97–2.40	0.071
Prior neoadjuvant/adjuvant trastuzumab	0.60	0.33–1.10	0.097	0.38	0.18–0.82	0.014
Cardiotoxicity during prior neoadjuvant/adjuvant treatment with trastuzumab and/or anthracyclines	2.36	1.05–5.30	0.037	4.48	1.56–12.87	0.005
Cumulative anthracycline exposure (total number of courses)^b^	1.05	0.98–1.14	0.155	1.05	0.97–1.15	0.247
Adjuvant radiotherapy						
No	Ref					
Left side	1.01	0.62–1.65	0.977			
Right side	1.00	0.57–1.73	0.987			
Side unknown	1.22	0.57–2.62	0.615			
De novo metastatic breast cancer	0.78	0.48–1.26	0.313	0.81	0.47–1.40	0.451

*PH* proportional hazards, *HR* hazard ratio, *CI* confidence interval, *BMI* body mass index, *LVEF* left ventricle ejection fraction, *MBC* metastatic breast cancer, *REF* reference category

^a^Based on multiple imputations with MICE of diabetes mellitus, hypertension, smoking, history of cardiac disease, local radiotherapy of the breast and prior cardiotoxicity during treatment with trastuzumab or anthracyclines, where death is a censoring event

^b^A course consist of doxorubicin 60 mg/m^2^ or epirubicin 100 mg/m^2^

Risk factors independently associated with severe cardiotoxicity were smoking (aHR 6.15, 95% CI 2.12–17.82), baseline LVEF < 60% (aHR 7.64, 95% CI 1.70–34.43) and cardiotoxicity during prior neoadjuvant/adjuvant treatment with trastuzumab and/or anthracycline (aHR 5.60, 95% CI 1.03–30.42, Table 3).

**Table 3:** Risk factors present at start of trastuzumab treatment for MBC associated with developing severe cardiotoxicity

	Univariable Cox PH^a^			Multivariable Cox PH^a^		
	HR	95% CI	*p*-value	Adjusted HR	95% CI	*p*-value
Age (years)	1.00	0.97–1.04	0.926			
BMI (kg/m^2^)						
< 25	Ref					
25–30	1.02	0.76–2.17	0.975			
> 30	1.99	1.13–3.73	0.188			
Hypertension	1.39	0.57–3.35	0.468			
Diabetes mellitus	0.65	0.09–4.82	0.673			
Smoking	6.61	2.23–19.60	< 0.001	6.15	2.12–17.82	< 0.001
History of cardiac disease	2.61	0.98–6.96	0.056			
Baseline LVEF(%)						
≥ 60	Ref					
< 60	9.91	2.34–42.05	0.002	7.64	1.70–34.43	0.008
Prior neoadjuvant/adjuvant trastuzumab	1.07	0.40–2.87	0.891			
Cardiotoxicity during prior neoadjuvant/adjuvant treatment with trastuzumab and/or anthracycline	3.30	0.87–12.56	0.079	5.60	1.03–30.42	0.045
Cumulative anthracycline exposure (total number of courses)^b^	1.21	1.07–1.37	0.003	1.15	1.00–1.33	0.051
Adjuvant radiotherapy						
No	Ref					
Left side	1.01	0.41–2.53	0.975			
Right side	0.73	0.23–2.31	0.597			
Unknown side	0.56	0.07–4.34	0.577			
De novo metastatic breast cancer	0.46	0.16–1.34	0.153			

*PH* proportional hazards, *HR* hazard ratio, *CI* confidence interval, *BMI* body mass index, *LVEF* left ventricle ejection fraction, *MBC* metastatic breast cancer, *REF* reference category

^a^Based on multiple imputations with MICE diabetes mellitus, hypertension, smoking, history of cardiac disease, local radiotherapy of the breast and prior cardiotoxicity during treatment with trastuzumab or anthracyclines, where death is a censoring event

^b^A course consist of doxorubicin 60 mg/m^2^ or epirubicin 100 mg/m^2^

Cause-specific Cox PH analyses, taking competing risk between cardiotoxicity and death into account, showed similar risk factors associated with non-severe + severe cardiotoxicity and severe cardiotoxicity (Supplementary Table S2).

LMM analysis (Table 4) also showed similar risk factors that were associated with LVEF differences over time as the Cox PH analyses (Tables 2 and 3). This means that patients who smoked on average had 2.77%-points lower LVEFs at the same time point compared to patients who did not smoke (Table 4; *p* < 0.001).

**Table 4:** Risk factors present at start of trastuzumab for MBC associated with continuous LVEF differences at each time point during total follow-up

	Estimated absolute LVEF difference at each time point (%)^a^	95% CI	Tail-probability
Cumulative trastuzumab exposure (months)^b^	0.27	− 0.06 to 0.51	0.163
Age (years)	0.02	− 0.05 to 0.09	0.642
BMI (kg/m^2^)			
< 25	Ref		
25–30	− 1.04	− 2.90 to 0.73	0.237
> 30	− 1.09	− 3.44 to 1.30	0.389
Hypertension	0.67	− 1.13 to 2.47	0.461
Diabetes mellitus	− 0.71	− 3.62 to 2.14	0.624
Smoking	− 2.77	− 5.26 to − 0.66	0.013
History of cardiac disease	0.40	− 2.21 to 3.04	0.766
Baseline LVEF (%)			
≥ 60	Ref		
< 60	− 6.72	− 8.17 to − 5.28	< 0.001
Prior neoadjuvant/adjuvant trastuzumab	2.11	− 0.09 to 4.31	0.060
Cardiotoxicity during prior neoadjuvant/adjuvant treatment with trastuzumab and/or anthracyclines	− 13.70	− 22.64 to − 5.45	< 0.001
Cumulative anthracycline exposure (total number of courses)^c^	− 0.38	− 0.69 to 0.08	0.012
Adjuvant radiotherapy			
No	Ref		
Left side	0.20	− 1.61 to 2.02	0.827
Right side	0.44	− 1.54 to 2.43	0.662
Side unknown	1.46	− 1.38 to 4.26	0.307
De novo metastatic breast cancer	1.39	− 0.45 to 3.24	0.144

*LVEF* left ventricle ejection fraction, *CI* credible interval, *BMI* body mass index, *MBC* metastatic breast cancer, *REF* reference category

^a^Based on multiple imputations with MICE of diabetes mellitus, hypertension, smoking, history of cardiac disease, local radiotherapy of the breast and prior cardiotoxicity during treatment with trastuzumab or anthracyclines

^b^From start of palliative trastuzumab treatment to each LVEF measurement

^c^A course consist of doxorubicin 60 mg/m^2^ or epirubicin 100 mg/m^2^

### Cumulative incidence of cardiotoxicity per relevant risk factors

**Fig. 2:**
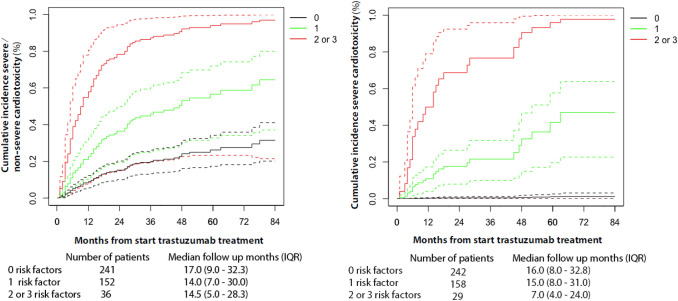
Cumulative incidence of non-severe + severe and severe cardiotoxicity with respect to the number of relevant risk factors. *Note* Solid lines indicate cumulative incidence; dashed lines indicate corresponding 95% CI of the cumulative incidence. For non-severe + severe cardiotoxicity, the following risk factors were included: BMI > 30 kg/m^2^, smoking and cardiotoxicity during prior neoadjuvant/ adjuvant treatment with trastuzumab and/or anthracyclines (Table 2). For severe cardiotoxicity, the following risk factors were included: smoking, cardiotoxicity during prior neoadjuvant/adjuvant treatment with trastuzumab and/or anthracyclines and baseline LVEF < 60% (Table 3)

The identified significant risk factors from the Cox PH analyses (Tables 2 and 3) were used to identify a patient group at low risk for cardiotoxicity, regardless of their effect size. In total, 241 patients had no relevant risk factors, 152 patients 1 risk factor and 36 patients 2 or 3 risk factors. Regarding relevant risk factors for severe cardiotoxicity, 242 had no risk factors, 158 patients 1 risk factors and 29 patients 2 or 3 risk factors. Patients without relevant risk factors for severe cardiotoxicity had a low cumulative incidence of 3.1% after a total follow-up of 4 years (Fig. 2). The cumulative incidence for both non-severe + severe cardiotoxicity and severe cardiotoxicity increases in case of more relevant risk factors.

### Sensitivity analyses evaluating detection bias of cardiotoxicity

Patients who received 0–2 LVEF measurements annually had lower risk of non-severe + severe cardiotoxicity and severe cardiotoxicity compared to patients who received 3–4 LVEF measurements annually (HR 0.43, 95% CI 0.24–0.78), however not of severe cardiotoxicity (HR 0.66, 95% CI 0.22–1.97). Additionally, patients who received > 4 LVEF measurements annually had higher risk of non-severe + severe cardiotoxicity (HR 6.39, 95% CI 3.57–11.41) and severe cardiotoxicity (HR 3.65, 95% CI 1.38–9.62).

### Reversibility of non-severe + severe cardiotoxicity and severe cardiotoxicity

**Fig. 3:**
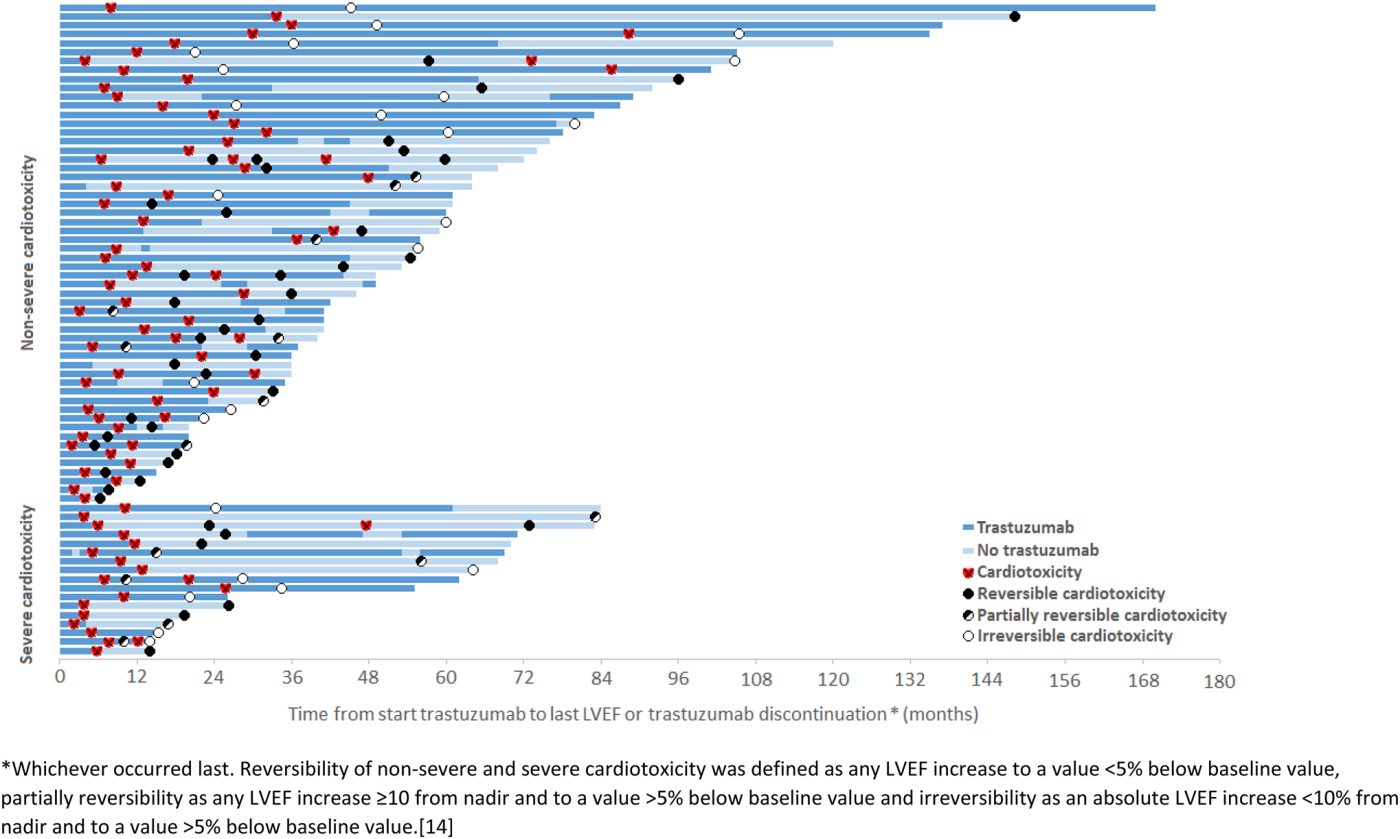
Swimmers plot of all patients who developed cardiotoxicity and received LVEF measurements thereafter (*n* = 74).

To explore the reversibility of cardiotoxicity, we categorized the patients who developed non-severe + severe cardiotoxicity into non-severe cardiotoxicity (*n* = 69) and severe cardiotoxicity (*n* = 25), of whom reversibility could be analysed in 58 patients with non-severe cardiotoxicity and 16 patients with severe cardiotoxicity (Fig. 3).

In total, the LVEF decline was reversible in 34 out of 58 patients (59%) who developed non-severe cardiotoxicity (Fig. 3 and Supplementary Figure S2). Among the 15 patients who discontinued trastuzumab for at least one cycle after non-severe cardiotoxicity, 7 patients received LVEF measurements. Of those patients, the LVEF was reversible in 4 patients (57%), partially reversible in 1 patient (14%) and irreversible in 2 patients (29%). Of the 43 patients who continued trastuzumab, 23 (56%) had normalization of LVEF, in 6 (15%) the LVEF decline was partially reversible and in 12 (29%) irreversible. Of all patients developing non-severe cardiotoxicity, 23% had cardiac symptoms including shortness of breath or angina pectoris. The reversibility was independent of the presence of cardiac symptoms.

The LVEF decline was reversible in 6 out of the 16 patients (35%) who developed severe cardiotoxicity and received LVEF measurements (Fig. 3 and Supplementary Figure S2). Among the 19 patients who discontinued trastuzumab after severe cardiotoxicity, the LVEF decline was reversible in 4 patients (40%), partially reversible in 3 patients (30%) and irreversible in 3 patients (30%). Among the 6 patients who continued trastuzumab after severe cardiotoxicity, the LVEF decline was reversible in 2 patients (33%), partially reversible in 3 patients (50%) and irreversible in 1 patient (17%). In the five patients with reversible severe cardiotoxicity, trastuzumab could be continued safely without recurring cardiotoxicity for a mean duration of 17 months (range 3–35). Of all patients who developed severe cardiotoxicity, 72% had cardiac symptoms. The reversibility was independent of the presence of cardiac symptoms.

## Discussion

In this study, we showed that among patients with HER2-positive MBC the yearly incidence of non-severe + severe cardiotoxicity was highest in the first 2 years of trastuzumab (11.7% and 9.1%, respectively) and gradually decreased over time. The median time to develop non-severe + severe cardiotoxicity was 11 months. The yearly incidence of severe cardiotoxicity was low over time (range 1.2–2.8%) with a median time to develop severe cardiotoxicity of 10 months. In non-smoking patients with baseline LVEF > 60% and no cardiotoxicity during prior neoadjuvant/adjuvant treatment (i.e. no relevant risk factors), the cumulative incidence of severe cardiotoxicity was limited to 3.1% after 4 years. Physicians often continued trastuzumab treatment despite cardiotoxicity, i.e. 62% in case of non-severe cardiotoxicity and 24% in case of severe cardiotoxicity. Interestingly, reversibility was relatively high among those who continued trastuzumab (71% after non-severe cardiotoxicity and 83% after severe cardiotoxicity of whom 56% and 33% fully recovered, respectively). Taken together, our data show the limited clinical relevance of regular cardiac monitoring by LVEF measurements in patients without relevant risk factors, stressing the need for an alternative monitor schedule.

The incidence of cardiotoxicity observed in this study was lower than reported in some other studies investigating cardiotoxicity of trastuzumab in the advanced setting [3, 7, 18]. This might be explained by the fact that these studies included patients treated with concomitant and higher doses of anthracyclines than our study [3, 18]. In addition, some studies used other imaging modalities besides MUGA scan and had a less strict criteria to define cardiotoxicity [7, 18].

We aimed to identify subgroups with particular low risk of developing cardiotoxicity in order to tailor cardiac monitoring. In our study, high BMI, smoking, cardiotoxicity during prior neoadjuvant/adjuvant treatment with trastuzumab and/or anthracyclines and baseline LVEF < 60% were found to be statistically significant independent risk factors for cardiotoxicity, whereas other cardiovascular risk factors were not. This might be due to the fact that our study population consisted of relatively young patients (median age was 54 years) with a good LVEF (> 50%) before starting trastuzumab. In the study of Rossi et al., age was an important risk factor for cardiotoxicity among patients receiving trastuzumab for HER2-positive MBC [8]. In the study of Guarneri et al., baseline LVEF and time from last anthracycline administration were important risk factors [7]. In addition, a recent study indicated that polymorphism HER2-Ile655 A > G is a risk factor for developing cardiotoxicity during trastuzumab [19]. Whether this risk factor will help personalizing cardiac monitoring in patients with HER2-positive MBC remains to be investigated.

By combining the incidence of cardiotoxicity over time with an individual cardiovascular risk profile, a tailored cardiac monitoring recommendation could be given, in case a yearly incidence of severe cardiotoxicity of less than 1%, while on average in women aged ≥ 50 in Europe the yearly incidence of heart failure ranges between 0.2 and 2.2% [20], is considered acceptable. This would result in the following recommendations. First, for patients with a baseline LVEF above 60%, without cardiotoxicity during prior neoadjuvant/adjuvant treatment with trastuzumab and/or anthracycline and who do not smoke, further serial cardiac monitoring during trastuzumab treatment could be omitted, since the cumulative incidence of severe cardiotoxicity in these patients after 4 years of trastuzumab treatment is low (3.1%). Second, for patients with ≥ 1 risk factor, cardiac monitoring during the first 3 years would be recommended as the yearly increase and absolute numbers of non-severe + severe cardiotoxicity were low after 3 years. Thereafter, cardiac monitoring could be performed in case of cardiac symptoms. The high reversibility rates of cardiotoxicity in a substantial number of patients with MBC, even after continuing trastuzumab, support our proposed individualized LVEF monitoring scheme during (long-term) trastuzumab treatment.

We did not have sufficient data on the use or start of cardio-protective medication which could be of value in determining trastuzumab continuation after cardiotoxicity and in evaluating the reversibility of cardiotoxicity. Furthermore, we could not assess whether the use or start of cardio-protective medication could be helpful for the proposed individualized LVEF monitoring schemes. Medication, including ACE inhibitors (perindopril, lisinopril), beta-blockers (carvedilol, bisoprolol) or angiotensin receptor blocker (candesartan), has shown to attenuate LVEF declines and thereby potentially increase the reversibility of LVEF declines [21–25].

To the best of our knowledge, this study is the largest in number with the longest follow-up duration investigating the reversibility of cardiotoxicity during trastuzumab in patients with HER2-positive MBC. Although the historical observational design of this cohort study provided a valuable opportunity to investigate clinical practice, some limitations inherent to historic cohorts should be mentioned. First, not all variables could retrospectively be collected and therefore not all variables, for example medication use and total dose of anthracycline treatment (mg/m^2^) and radiation treatment (Gy), could be investigated. Although some missing data were observed for other variables as well, these variables could be used after multiple imputation as sensitivity analysis with complete case analysis showed similar risk factors associated with non-severe + severe cardiotoxicity as after multiple imputations (Supplementary Table S3). Second, due to the lack of clear cardiac monitoring guidelines, the timing of the LVEF measurements was not standardized but chosen by the treating physician. Therefore, ascertainment of cardiotoxicity could be delayed and detection bias cannot be excluded. However, information bias cannot have influenced the incidence of cardiotoxicity as LVEF measurements after cardiotoxicity were not taken into account. Third, most LVEFs (83%) were measured by MUGA scanning with a known high inter-observer and intra-observer variability in measuring the LVEF [26, 27]. However, by using a LMM analysis that takes into account all available LVEF measurements, the effect of this variability is minimized. Fourth, in estimating the cumulative incidence of cardiotoxicity per number of relevant risk factors, the effect size of the individual risk factors was not taken into account. Fifth, as the primary endpoint of the study was the development of cardiotoxicity, we did not investigate other cardiac co-morbidities that could develop over time. In addition, due to the median follow-up of the study of 15 months, we were unable to assess this. Last, only 26 patients (6%) in our cohort received dual HER2-targeted therapy, which is the current standard of first-line treatment for patients with HER2-positive MBC. However, as pertuzumab does not increases the risk of cardiotoxicity [28], the results of this study are likely to be applicable to daily clinical practice.

## Conclusion

The cumulative incidence of severe cardiotoxicity in non-smoking patients with a baseline LVEF above 60% who had no cardiotoxicity during prior neoadjuvant/adjuvant treatment with trastuzumab and/or anthracycline after 4 years of trastuzumab treatment is low (3.1%). Therefore, serial cardiac monitoring can be omitted in these patients during long-term trastuzumab use.

## Supplementary Information

Below is the link to the electronic supplementary material.

Electronic supplementary material 1 (DOCX 199 kb)
