# Supplementary material for: Cardiotoxicity during long-term trastuzumab use in patients with HER2-positive metastatic breast cancer: who needs cardiac monitoring?
Source: Breast Cancer Res Treat. 2021 Jan 4;186(3):851–62. doi: 10.1007/s10549-020-06039-w (PMC8019427; doi:10.1007/s10549-020-06039-w)
Supplement: Supplementary file 1 — Electronic supplementary material 1 (DOCX 199 kb) [file 10549_2020_6039_MOESM1_ESM.docx]

**Supplementary material**

Supplementary material to Bouwer NI, Steenbruggen TG, van Rosmalen J et al. **Cardiotoxicity during long-term trastuzumab use in HER2-positive metastatic breast cancer patients: who needs cardiac monitoring?**

**Supplementary Fig S1** Flowchart of included patients


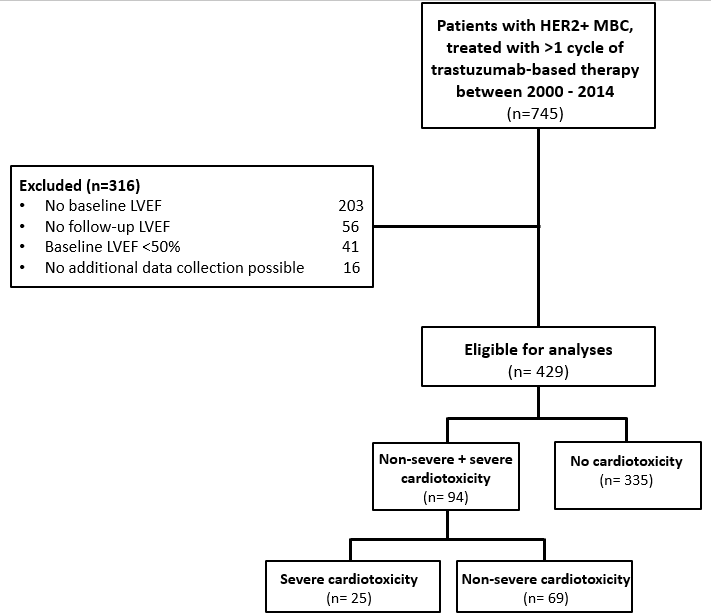


***Abbreviations*** *HER2+, Human Epidermal growth factor Receptor 2 positive; MBC, metastatic breast cancer; LVEF, left ventricle ejection fraction*

**Supplementary Table S1** Differences in risk factors present at start of trastuzumab for MBC between included and excluded patients

|  | **Included patients n=429 (%)** | **Excluded patients n=339 (%)** | **P-value** |
| --- | --- | --- | --- |
| Age (years) | 53.8 | 53.5 | 0.699 |
| BMI(kg/m^2^)  <25   1. – 30   >30 | 164 (38)  130 (30)  44 (10) | 77 (23)  73 (22)  32 (9) | 0.252 |
| Hypertension | 98 (23) | 60 (18) | 0.928 |
| Diabetes mellitus | 28 (7) | 19 (6) | 0.637 |
| Smoking | 90 (12) | 53 (16) | 0.502 |
| History of cardiac disease | 35 (8) | 26 (8) | 0.373 |
| Baseline LVEF(%)^a^  ≥60  <60 | 198 (46)  231 (54) | 42 (53)  38 (48) | 0.297 |
| Prior neoadjuvant/adjuvant trastuzumab | 84 (20) | 47 (14) | 0.038 |
| Cardiotoxicity during prior neoadjuvant/adjuvant treatment with trastuzumab and/or anthracyclines | 15 (3) | 18 (5) | 0.283 |
| Cumulative anthracycline exposure (total number of courses) | 3.0 | 3.1 | 0.646 |
| Adjuvant radiotherapy  No  Left side  Right side  Side unknown | 171 (40)  126 (29)  100 (23)  32 (7) | 152 (45)  81 (24)  68 (20)  37 (11) | 0.084 |
| De novo MBC | 118 (28) | 86 (25) | 0.506 |

***Abbreviations*** *BMI, body mass index; LVEF, left ventricle ejection fraction*

^a^ 80 baseline LVEF were measured in the excluded patients

^b^ A course consist of doxorubicine 60 mg/m^2^ or epirubicine 100 mg/m^2^

**Supplementary Table S2** Risk factors present at start of trastuzumab for MBC associated with non-severe + severe cardiotoxicity and severe cardiotoxicity when adjusted for death

|  | Non-severe + severe cardiotoxicity | | | Severe cardiotoxicity | | |
| --- | --- | --- | --- | --- | --- | --- |
|  | **AdjustedHR** | **95% CI** | **P-value** | **Adjusted HR** | **95% CI** | **P-value** |
| Age (years) | 1.01 | 0.99 – 1.03 | 0.235 | NA |  |  |
| BMI (kg/m^2^)  <25  25-30  >30 | REF  1.24  1.85 | 0.71 – 2.15  0.96 – 3.58 | 0.447  0.066 | NA  NA |  |  |
| Smoking | 2.12 | 1.21 – 3.72 | 0.008 | 2.14 | 1.23 – 3.73 | 0.007 |
| Baseline LVEF(%)  ≥60  <60 | REF  1.44 | 0.90 – 2.29 | 0.125 | 1.43 | 0.91 – 2.24 | 0.122 |
| Prior neoadjuvant/adjuvant trastuzumab | 0.38 | 0.16 – 0.92 | 0.030 | NA |  |  |
| Cardiotoxicity during prior neoadjuvant/adjuvant treatment with trastuzumab and/or anthracycline | 5.37 | 1.30 – 22.12 | 0.017 | 2.93 | 1.18 – 7.30 | 0.019 |
| Cumulative anthracycline exposure (total number of courses)^a^ | 1.05 | 0.96 – 1.15 | 0.263 | 1.03 | 0.95 – 1.12 | 0.505 |
| De novo metastatic breast cancer | 0.85 | 0.50 – 1.47 | 0.571 | NA |  |  |

***Abbreviations*** *HR, hazard ratio; CI, confidence interval; BMI, body mass index; LVEF, left ventricle ejection fraction; MBC, metastatic breast cancer; REF, reference category*

Multivariable cause-specific Cox proportional hazard regression analysis with multiple imputations with substantive model compatible version of fully conditional specification (SMC-FCS) of diabetes mellitus, hypertension, smoking, history of cardiac disease, local radiotherapy of the breast and cardiotoxicity during prior neoadjuvant or adjuvant treatment with trastuzumab and/or anthracycline

^a^ A course consist of doxorubicine 60 mg/m^2^ or epirubicine 100 mg/m^2^

**Supplementary Figure S2** Reversibility of cardiotoxicity subdivided to the physicians’ decision of trastuzumab (dis)continuation

**
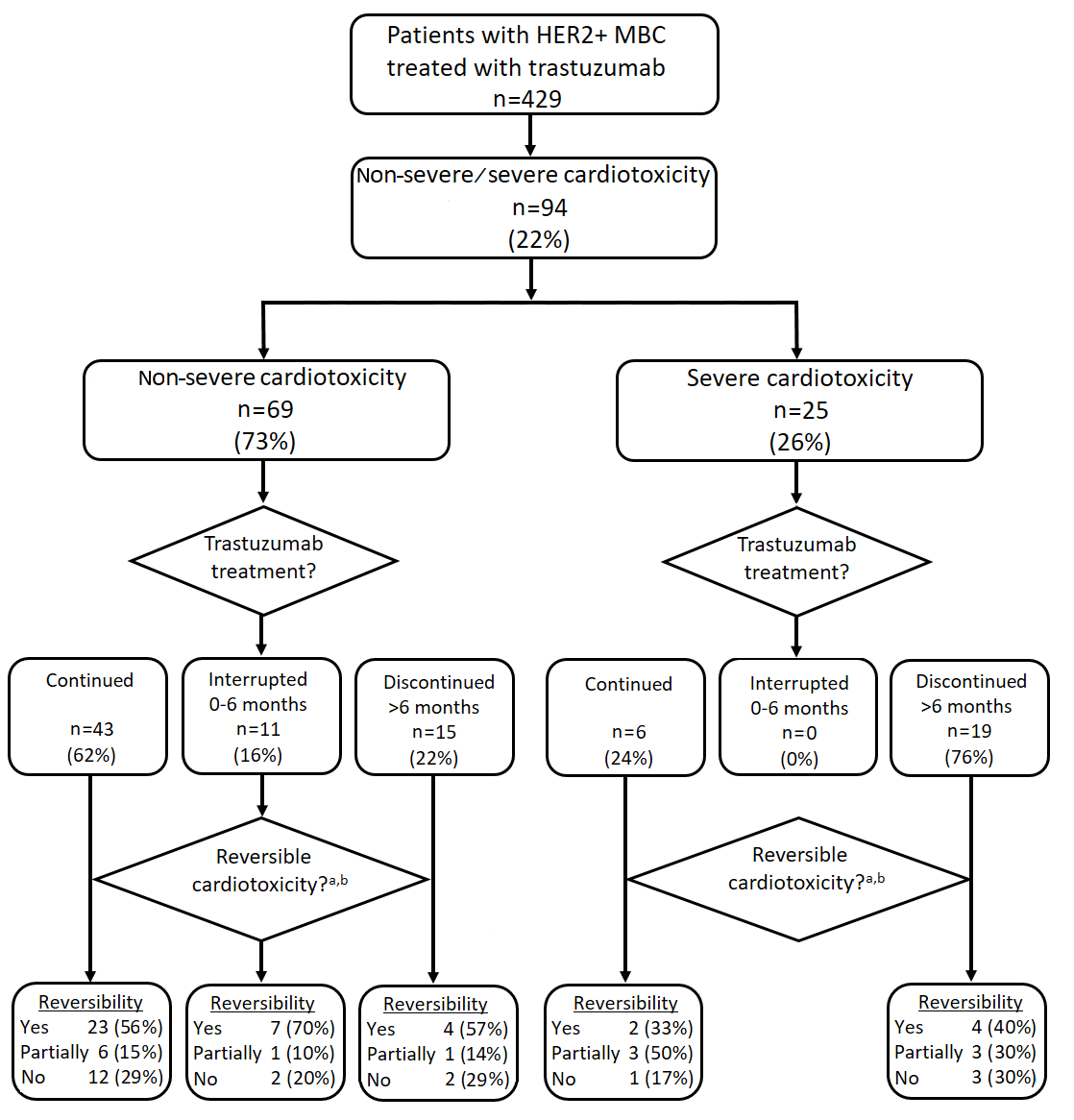
**

***Abbreviations*** *HER2+, Human Epidermal growth factor Receptor 2 positive; MBC, metastatic breast cancer.*

^a^ After development of cardiotoxicity, no LVEF measurements were available for some patients. Therefore, the reversibility of cardiotoxicity could not be assessed for every patient and the percentage is taken from the patients that received cardiac monitoring.

^b^ Reversibility of non-severe and severe cardiotoxicity was defined as any LVEF increase to a value <5% below baseline value, partially reversibility as any LVEF increase ≥10 from nadir and to a value >5% below baseline value and irreversibility as an absolute LVEF increase <10% from nadir and to a value >5% below baseline value.[14]

**Supplementary Table S3** Sensitivity analysis with complete case analysis of risk factors present at start of trastuzumab treatment for MBC associated with non-severe + severe cardiotoxicity

|  | Univariable Cox PH^a^ | | | Multivariable Cox PH^a^ | | |
| --- | --- | --- | --- | --- | --- | --- |
|  | **HR** | **95% CI** | **P-value** | **Adjusted HR** | **95% CI** | **P-value** |
| Age (years) | 1.01 | 0.99 – 1.02 | 0.579 | 1.01 | 0.98 – 1.04 | 0.574 |
| BMI (kg/m^2^)  <25  25-30  >30 | REF  1.07  1.79 | 0.63 – 1.81  0.94 – 3.42 | 0.810  0.078 | 0.80  2.00 | 0.38 – 1.68  0.88 – 4.53 | 0.547  0.096 |
| Hypertension | 1.21 | 0.77 – 1.92 | 0.414 |  |  |  |
| Diabetes mellitus | 1.11 | 0.49 – 2.56 | 0.790 |  |  |  |
| Smoking | 1.88 | 1.13 – 3.16 | 0.013 | 2.32 | 1.20 – 4.47 | 0.012 |
| History of cardiac disease | 1.34 | 0.69 – 2.58 | 0.389 |  |  |  |
| Baseline LVEF(%)  ≥60  <60 | REF  1.60 | 1.05 – 2.44 | 0.031 | 2.06 | 1.01 – 4.18 | 0.046 |
| Prior neoadjuvant/adjuvant trastuzumab^b^ | 0.60 | 0.33 – 1.10 | 0.098 | 0.89 | 0.32 – 2.43 | 0.814 |
| Cardiotoxicity during prior neoadjuvant/adjuvant treatment with trastuzumab and/or anthracycline | 3.60 | 1.65 – 7.68 | 0.001 | 6.76 | 2.10 – 21.73 | 0.001 |
| Cumulative anthracycline exposure (total number of courses)^b^ | 1.06 | 0.98 – 1.14 | 0.157 | 1.09 | 0.95 – 1.24 | 0.216 |
| Adjuvant radiotherapy  No  Left side  Right side  Side unkown | REF  1.01  1.00  1.22 | 0.62 – 1.65  0.57 – 1.73  0.57 – 2.62 | 0.975  0.986  0.613 |  |  |  |
| De novo metastatic breast cancer | 1.28 | 0.79 – 2.06 | 0.315 | 0.91 | 0.41 – 2.02 | 0.815 |

***Abbreviations*** *PH, proportional hazards; HR, hazard ratio; CI, confidence interval; BMI, body mass index; LVEF, left ventricle ejection fraction; MBC, metastatic breast cancer; REF, reference category*

^a^ Not based on multiple imputations of diabetes mellitus, hypertension, smoking, history of cardiac disease, local radiotherapy of the breast and prior cardiotoxicity during treatment with trastuzumab or anthracyclines but based on only the complete cases

^b^ A course consist of doxorubicine 60 mg/m^2^ or epirubicine 100 mg/m^2^
